# Structural materials with afterglow room temperature phosphorescence activated by lignin oxidation

**DOI:** 10.1038/s41467-022-33273-1

**Published:** 2022-09-20

**Authors:** Keliang Wan, Bing Tian, Yingxiang Zhai, Yuxuan Liu, He Wang, Shouxin Liu, Shujun Li, Wenpeng Ye, Zhongfu An, Changzhi Li, Jian Li, Tony D. James, Zhijun Chen

**Affiliations:** 1 Engineering Research Center of Advanced Wooden Materials and Key Laboratory of Bio-based Material Science & Technology, Northeast Forestry University, Ministry of Education, Harbin, China; 2 CAS Key Laboratory of Science and Technology on Applied Catalysis, Dalian Institute of Chemical Physics, Chinese Academy of Sciences, Dalian, China; 3 Key Laboratory of Flexible Electronics & Institute of Advanced Materials, Nanjing Tech University, Nanjing, China; 4 Department of Chemistry, University of Bath, Bath, BA2 7AY United Kingdom

**Keywords:** Optical materials, Synthesis and processing, Bioinspired materials

## Abstract

Sustainable afterglow room temperature phosphorescence (RTP) materials, especially afterglow RTP structural materials, are crucial but remain difficult to achieve. Here, an oxidation strategy is developed to convert lignin to afterglow materials with a lifetime of ~ 408 ms. Specifically, lignin is oxidized to give aromatic chromophores and fatty acids using H_2_O_2_. The aromatic chromophores are locked by a fatty acid-based matrix by hydrogen bonds, triggering enhanced spin orbit coupling and long afterglow emission. More interestingly, motivated by this discovery, an auto fabrication line is built to convert wood (natural structural materials) to wood with afterglow RTP emission (RTP wood) via in situ oxidation of naturally-occurring lignin located in the wood cell walls to oxidized lignin (OL). The as-prepared RTP wood exhibits great potential for the construction of sustainable afterglow furniture. With this research we provide a new strategy to promote the sustainability of afterglow RTP materials and structural materials.

## Abstract (web summary)

Sustainable afterglow room temperature phosphorescence (RTP) Structural materials are difficult to achieve. Here, the authors demonstrate a wood based RTP material by oxidation of lignin to realize an afterglow RTP material with a lifetime of ~ 408 ms.

## Introduction

Plant biomass is a potential source of renewable chemicals and materials^1,2^. Lignin, one of its main components accounting for 15–30% of the total mass, forms via the oxidative polymerization of p-hydroxycinnamyl alcohol monolignols coming from the phenylpropanoid pathway^3^. Additionally, technical lignin is produced as a byproduct of pulp and paper industry in very large quantities (~60–70 Mt per year)^4,5^. The need for lignin valorization has become well understood in the scientific community, which has given rise to a large number of studies being published over the last several years^6,7^. From a chemical perspective, lignin mainly consists of *p*-hydroxyphenyl (H), guaiacyl (G) and syringyl (S) units, linked primarily by *β*-O-4 and C-C bonds^8,9^. This chemical structure endows lignin with great potential as for a core component for functional materials and aromatic compounds^10–12^. Moreover, lignin exhibited interesting bioactivity, facilitating applications in biomedicine, agriculture, and biomass conversion^13,14^. Attributed to the incorporated aromatic structures, lignin can generate interesting photo physicochemical properties^15,16^. Recently, our group demonstrated that afterglow RTP emission of lignin can be achieved through encapsulation in a polyacrylic acid matrix^17^.

Afterglow RTP materials have wide applications in electronic devices, optical sensing, biological imaging and information encryption^18,19^. To achieve effective RTP, two crucial conditions should be satisfied. First, the triplet excitons could be effectively populated by facilitating ISC from S_1_ to T_n_. Second, the nonradiative deactivation of triplet excitons is suppressed and the radiative transition is promoted from the lowest excited triplet state (T_1_) to the ground state (S_0_)^20^. Until now, small molecules, polymers, supramolecules, carbon dots and MOFs have been reported for the effective afterglow RTP emission^21,22^. In particular, preparing afterglow RTP materials from natural sources, are particularly sought after since natural sources are abundant, sustainable, flexible and biocompatible^23,24^. Nevertheless, two main challenges remain: (1) Converting sustainable lignin to afterglow RTP materials requires the use of a petrol-derived matrix (~95 w/w%), which does not meet the requirements for a sustainable system^17^. (2) Most of afterglow RTP materials, including lignin-derived afterglow RTP materials, exist as powders, crystals, films, liquids or porous materials^25–32^. However, very few afterglow RTP structural materials have been reported, yet they exhibit high mechanical performance and are crucial in materials science and technology^33^.

**Fig. 1:**
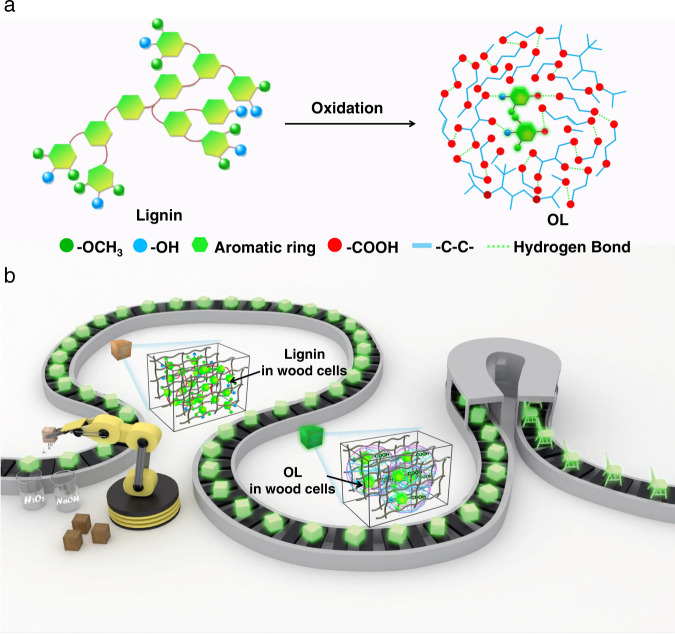
Schematic illustrating of lignin oxidation for RTP. **a** Design of afterglow materials from lignin via oxidation strategy. **b** Preparation of RTP wood and furniture from natural wood via an automatic line.

Here, we developed an oxidation method for converting lignin to sustainable afterglow RTP materials without the addition of additional synthetic matrix. Specifically, the G units and S units of lignin were oxidized to give G acids and S acids (chromophores), which are then in situ locked by fatty acids (as matrix, also due to lignin oxidation) via hydrogen bonding (Fig. 1a). As a result, the OL exhibits efficient afterglow emission. More interestingly, motivated by this discovery, an auto fabrication line was built to convert wood, a natural structural material, to RTP wood via in situ oxidation of naturally occurring lignin located in the wood cell walls to OL (Fig. 1b). The as-prepared RTP wood exhibited great potential for constructing afterglow sustainable furniture.

## Results

### RTP emission of OL

**Fig. 2:**
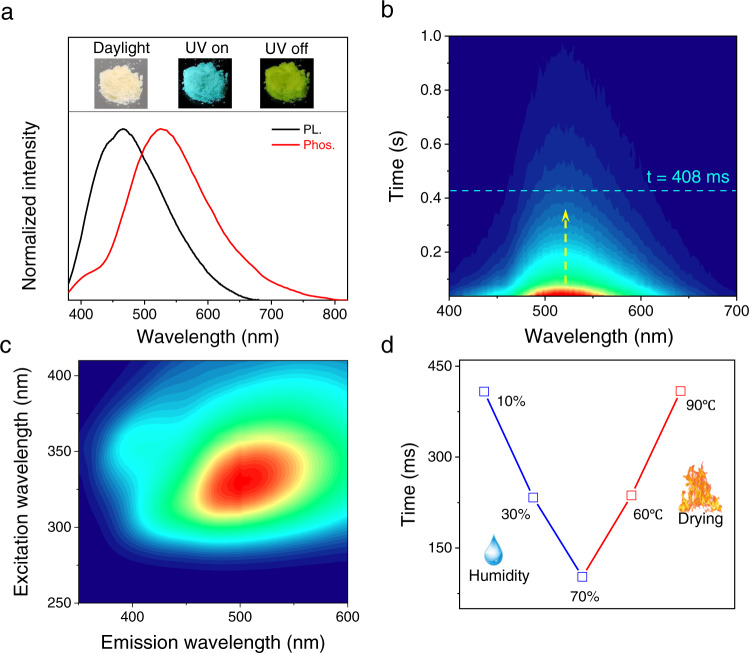
Afterglow RTP emission of OL. **a** Fluorescence (PL.) and RTP (Phos.) emission of OL, excitation wavelength = 365 nm, inset: Images of bright field, fluorescent and RTP emission of OL upon UV irradiation (365 nm). **b** Time-resolved RTP emission of OL, excitation wavelength = 365 nm. **c** Excitation-dependent RTP emission of OL; **d** RTP lifetime of OL upon exposure to humidity and drying.

The ultraviolet-visible (UV-Vis) spectra of OL (prepared from lignosulfonates, see the details in the methods) exhibits optical absorbance predominantly in the UV region, over the wavelength range from 200 nm to 400 nm (Supplementary Fig. 1). OL exhibited a decreased absorbance, compared to the raw lignin (Supplementary Fig. 2). Raw lignin and OL also exhibited obvious differences in the FT-IR spectra (Supplementary Fig. 3). The signal intensity of -C = O at 1704 cm^−1^ and -O-H at 3300 cm^−1^ increased after lignin oxidation. A decrease in the molecular weight of lignin after oxidation was observed using gel permeation chromatography (GPC) (Supplementary Fig. 4). The weight-average molecular weight of lignin decreased from 11541 to 1588 after oxidation. Thermogravimetric analysis (TGA) and derivative thermogravimetry (DTG) were also investigated before and after lignin oxidation (Supplementary Fig. 5). These results indicated that lignin degraded into small fragments after oxidation. Upon UV irradiation, OL exhibited strong fluorescence emission, centered at ~464 nm (Fig. 2a). Surprisingly, afterglow RTP emission was also observed, with phosphorescence centered at ~525 nm. Time-resolved spectroscopy indicated that the OL displays a long-lasting and stable afterglow emission and retained weak phosphorescence emission at the same emission wavelength for as long as 800 ms (Fig. 2b). The lifetime of OL was ~408 ms (Supplementary Fig. 6). Moreover, excitation-independent RTP emission of OL was observed. The RTP emission wavelength red shifted from 460 nm to 550 nm when the excitation wavelength changed from 275 nm to 400 nm, indicating multiple chromophores in OL (Fig. 2c). Additionally, to illustrate the generality of our strategy, another technical lignin, alkali lignin, was also treated using the same method to obtain OL. As expected, the as-prepared OL from alkali lignin produced efficient afterglow RTP emission with a lifetime of ~215 ms (Supplementary Fig. 7). The afterglow RTP was found to be humidity-sensitive, with reduction of the phosphorescence lifetime when the humidity was gradually increased. In particular, the phosphorescence lifetime dropped rapidly from ~408 ms to ~102 ms when the humidity increased from 10% to 70% (Fig. 2d). To understand the effect of humidity on the lifetime, OL was exposed to different levels of humidity. The results indicated that the lifetime of OL was completely quenched when the humidity reached 100% (Supplementary Fig. 8). More interestingly, this quenched lifetime was recovered again after drying the OL. The “humidity-drying” sensitive RTP lifetime was stable and it could be recycled 7 times (Supplementary Fig. 9). Notably, OL exhibited a decreased lifetime upon exposure to increased temperature (from 100 −175 ^o^C) for an extended time (~90 min) (Supplementary Fig. 10). It was possibly attributed to decomposition of lignin, as determined by TGA and DTG analysis. Additionally, OL displayed mechanoresponsive RTP. The afterglow RTP lifetime decreased from ~408 ms to ~248 ms when the external pressure increased from 0 MPa to 60 MPa (Supplementary Fig. 11). Additionally, the afterglow emission of OL was stable in organic solvents. The lifetimes were 489.34, 516.55 and 519.84 ms in DCM, ETOH and CH_3_CN, respectively (Supplementary Fig. 12).

### Mechanism investigation

**Fig. 3:**
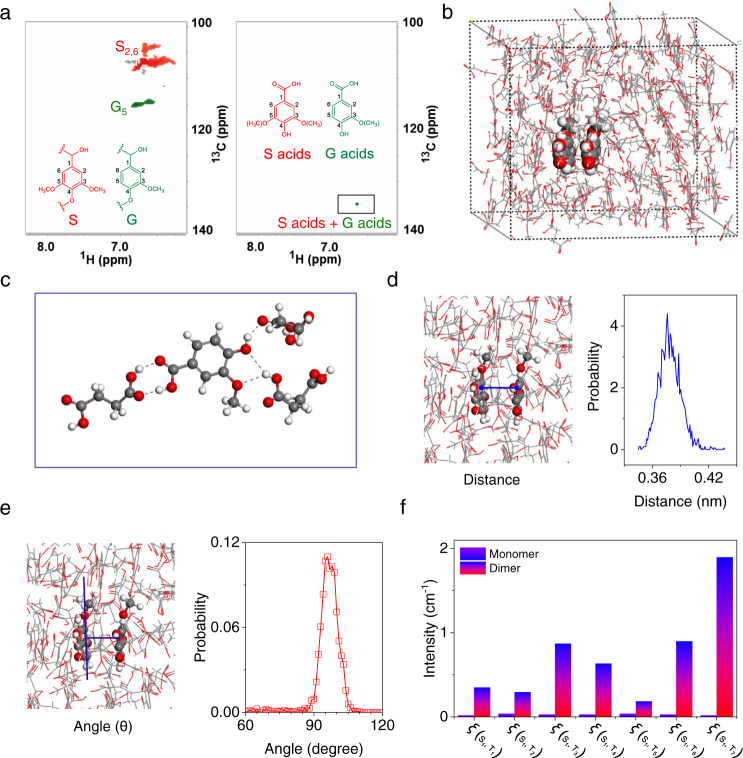
Mechanism of afterglow RTP of OL. **a** 2D HSQC NMR of lignin (left) and OL (right) in the aromatic region. **b** and **c**, Simulation of molecular interactions between model aromatic acids and fatty acids in OL. **d** Center-center distance between adjacent aromatic molecules in OL. **e**, Relative orientation of two neighboring aromatic model compounds in OL. **f** Spin orbit coupling ξ (S_1_, T_n_, *n* = 1, 2, 3…7) of vanillic acid dimers and vanillic acid monomer. In the Figure **b, c, d** and **e**, Red dot: oxygen atom, Black dot: carbon atom, White dot: hydrogen atom; Black line: chemical bond (-C-C/-C = C-); Dot line: hydrogen bond.

To gain a deeper insight into the mechanism of afterglow RTP emission of OL, a set of experiments was conducted. The 2D heteronuclear singular quantum correlation nuclear magnetic resonance (2D HSQC NMR) indicated that lignin mainly consists of G units and S units in the aromatic region and the ratio of G and S units was 10:90 (Fig. 3a). Lignin also displayed abundant signals between 3.0 ppm and 6.0 ppm, which was attributed to the C-O-C and C-C linkers (Supplementary Fig. 13). After oxidation, most of the signals in the aromatic region (6.2–6.8 ppm, 103–115 ppm) disappeared. In response, the signals of G acids and S acids were observed at 6.6 ppm and 134 ppm, which were exactly the oxidated products from G and S units, respectively. While, OL displayed new signals between 3.0 ppm and 6.0 ppm (Supplementary Fig. 13), probably from fatty acids. To further verify the structural evolution of lignin, high-resolution MS (HRMS) analysis was conducted on an OL sample. As expected, G/S acids were observed in the HRMS spectra (Supplementary Fig. 14). Moreover, fatty acids including sulfonic fatty acids and fatty carboxylic acids were detected, confirming that the 2D HSQC NMR signals between 3.0 and 6.0 ppm were due to fatty acids (Supplementary Fig. 14). Pyrolysis gas chromatography-mass spectrometry (Py-GC-MS) analysis was conducted to further understand the structural change. The results indicated that lignin had degraded into smaller fragments after oxidation (Supplementary Fig. 15 vs Supplementary Fig. 16). According to literature^34–36^, and as-obtained HRMS, Py-GC-MS, and GPC results, the proposed pathways for the oxidative depolymerization of lignin into aromatic acids and fatty acids are shown in Supplementary Fig. 17, which went through oxidative C_α_-C_β_ cleavage and oxidative ring-open reactions, respectively. All the above results indicate that OL contains both aromatic acids and fatty acids. Theoretical simulation further indicated that the aromatic acids (taking vanillic acid as a model compound) could be “locked” by fatty acids (taking succinic acid as a model compound) by hydrogen bonding in OL (Fig. 3b). The simulation results indicated that carbonyl moieties of the aromatic acids formed intermolecular hydrogen bonds, restricting vibration, which enhances spin-orbit coupling and encourages phosphorescence (Fig. 3c)^27^. Additionally, the results indicated that the average center-center distance between adjacent molecules was ca. ~0.37 nm, facilitating the formation of strong π - π interactions between the aromatic acids (Fig. 3d). The relative orientation of two neighboring model compounds (*θ*) was populated over a range from ~60^o^ to 100^o^ with an optimal angle of ~95^o^, indicating H-type dimers formed between them (Fig. 3e)^27,37^. Such H-type dimers can stabilize the lowest excited triplet states, and prolong the RTP lifetime. Moreover, the calculation result indicated that the spin-orbit coupling ξ (S_1_, T_n_) of the H-type dimers is larger than that of monomer molecule, facilitating phosphorescence (Fig. 3f). Additionally, urea, as a reagent for breaking hydrogen bonding and reducing the interaction between aromatic acids and fatty acids, was added to powdered OL^38^. Immediately, the lifetime of OL decreased to ~212 ms when the urea fraction increased to 28% (Supplementary Fig. 18). To demonstrate how the urea affected the OL, theoretical calculations were performed. The interaction force between model aromatic acids and fatty acids was 79.9 kJ/mol and the intermolecular interaction between fatty acids was 78.7 kJ/mol. However, the interaction between urea and fatty acids was 83.7 kJ/mol, which was higher than the interactions with OL and as such can break the hydrogen bonding between aromatic acids and fatty acids (Supplementary Fig. 19). Moreover, the hydrogen bond formed between fatty acids and urea was confirmed by a downfield shift of the carboxyl and amino protons in the 1H NMR (Supplementary Fig. 19). According to the literature^39^, hydrogen bonds can induce a decrease of electron density near the hydroxyl proton and deshield the nuclei, resulting in a downfield shift of the hydrogen-bonded protons. The above ^1^H NMR results provide direct experimental evidence for the formation of hydrogen bonds between succinic acid and urea. Additionally, humidity-responsive lifetimes could be attributed to decreased hydrogen bonding between the fatty acids and aromatic acids. This was confirmed by theoretical calculations. Water exhibits stronger binding energy with fatty acids (82.6 kJ/mol) than aromatic acid. As a result, the addition of water can break the hydrogen bonding between the fatty acids and aromatic acids, facilitating the free rotation of the carbonyl moieties and decreasing SOC and ISC. As such, the lifetime decreased upon exposure to high humidity. (Supplementary Fig. 19). Based on all the above results, a plausible mechanism was proposed. Firstly, the oxidation of lignin by H_2_O_2_ produced aromatic acids and fatty acids. After that, the aromatic acids became “locked” by fatty acid hydrogen bonds. Finally, the “locking” restricted the vibration of the carbonyl moieties and encouraged the formation of H-type dimers, which promoted spin-orbit coupling and afterglow emission.

### Activating RTP of natural wood

**Fig. 4:**
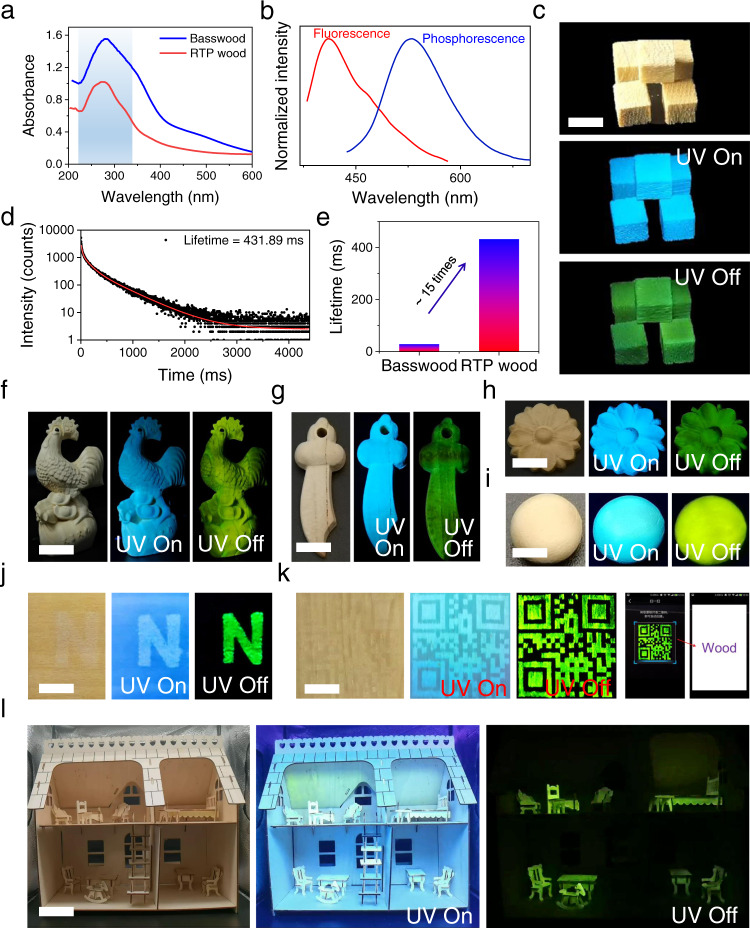
Preparation of RTP wood. **a** UV-Vis spectra of natural Basswood and corresponding RTP wood. **b** Fluorescence and phosphorescence of RTP wood made from Basswood. **c** RTP wood made from Basswood under daylight, 365 nm UV lamp on and off, scale bar = 1 cm. **d** Lifetime of RTP wood made from Basswood. **e** Comparison between lifetime of natural Basswood and RTP wood made from Basswood. **f** and **g**, RTP wood made from peach wood under daylight, 365 nm UV lamp on and off (scale bar = 1 cm in **f**; scale bar = 1 cm in g). **h** RTP wood made from Rubber wood under daylight, 365 nm UV lamp on and off (scale bar = 1 cm). **i** RTP wood made from Schima superba under daylight, 365 nm UV lamp on and off (scale bar = 1 cm). **j** and **k** Patterned RTP wood under daylight, 365 nm UV lamp on and off (scale bar = 0.5 cm in **j**; scale bar = 1 cm in **k**). **l**, Afterglow furniture made from RTP wood under daylight, 365 nm UV lamp on and off (scale bar = 4 cm).

Inspired by this discovery, naturally-occurring lignin in wood cell walls was oxidized in situ to generate OL to generate RTP wood. In addition, a smart manufacturing line for RTP wood was constructed. In this line, an automatic manipulator was used to control the immersion of the wood in NaOH and H_2_O_2_ solution (Supplementary Fig. 20). Specifically, wood was pretreated by NaOH solution to make the lignin in the cell walls more accessible^40^. After that, H_2_O_2_ solution facilitated in situ oxidation. Thanks to the smart manufacturing line, RTP wood was prepared efficiently (Supplementary Movie 1). UV-Vis spectra indicated that the absorbance intensity at 250-350 nm, was due to the absorbance of lignin^41^, which decreased significantly after in situ oxidation of the natural wood (Fig. 4a). The as-prepared RTP wood from Basswood after drying exhibited long afterglow RTP emission centered at 510 nm with a lifetime of ~431 ms (Figs. 4b-d). As a control, untreated Basswood displayed phosphorescence emission with a lifetime of 28.25 ms, ~15 times lower than that for RTP wood (Fig. 4e and Supplementary Fig. 21)^17^. Notably, the wood became more hydrophilic after oxidation, as demonstrated by the decrease in contact angle (from 108 to 55 degree) (Supplementary Fig. 22). Such changes led to a sensitive response of RTP wood to water. Immersing the wood in water immediately quenched its emission (Supplementary Fig. 23). However, the problem can be solved by coating RTP wood with hydrophobic wax. Such coated RTP wood exhibited afterglow emission when it was immersed in water (Supplementary Fig. 24). Moreover, the method for producing RTP wood was widely applicable. For example, different types of wood including peach wood, rubber wood, and Schima superba were efficiently converted to RTP woods using the in situ oxidation method (Fig. 4f-i and Supplementary Fig. 25–27). The lifetimes of RTP wood made from peach wood, rubber wood, and Schima superba increased from 19.53, 70.35 and 60.95 to 284.00, 197.31 and 224.91 ms, respectively (Supplementary Fig. 28). Interestingly, a patterned RTP wood could be achieved by the selective oxidation of the wood surface. As such N shape and 2-D code were patterned on natural wood. After switching off the UV light source, afterglow RTP images could be clearly observed (Fig. 4j, k). Moreover, the 2-D code could be recognized using a smartphone (Fig. 4k and Supplementary movie 2). As a practical demonstration, a series of model afterglow furniture were constructed using RTP wood (Fig. 4l). Upon light irradiation, the furniture made from RTP wood exhibited nice fluorescence. In addition, nice afterglow RTP was observed after switching off the light source. Considering the effect and importance of sustainable indoor lighting materials for the physical and psychological well-being of building occupants, our afterglow furniture has great potential for house decorations.

## Discussion

In summary, lignin was successfully converted to sustainable afterglow OL with a lifetime of ~408 ms using an oxidation strategy. More interestingly, motivated by this discovery, an auto fabrication line was built to convert natural wood to RTP wood using in situ oxidation of naturally occurring lignin located in the wood cell walls to generate OL. The RTP wood generated was then successfully processed into afterglow furniture. This work not only demonstrates the sustainability of afterglow RTP materials, but also provides new afterglow RTP structural materials. From a wider perspective, considering the sustainability and ease of processibility of natural wood, such RTP wood could have great potential for wood architecture and light management devices.

## Methods

### Preparation of OL

Sodium lignosulfonate (Alkali lignin) (2 g) was dispersed in H_2_O_2_ solution (10%, 120 mL). The mixture was heated in an oil bath at 120 °C for 8 h. The resulting solution was dried to give OL. The OL powder was then ground and dried at 120 °C for 30 min.

### Preparation of RTP Wood

Wood was firstly immersed in NaOH (0.15 g/mL) solution for 3 s. Subsequently, the wood was immersed in H_2_O_2_ (30%) for 5 s. The treated wood was then dried at 120 °C for 10 min.

## Supplementary information

Supplementary Information

Description of Additional Supplementary Files

Supplementary Movie 1

Supplementary Movie 2

## Data Availability

All relevant data are included in this article and its Supplementary Information files. Source data are provided with this paper.

## Source data

Source Data

## References

[1] Chen C, Berglund L, Burgert I, Hu L (2021). Wood nanomaterials and nanotechnologies. Adv. Mater..

[2] Droguet BE (2022). Large-scale fabrication of structurally coloured cellulose nanocrystal films and effect pigments. Nat. Mater..

[3] Neutelings G (2011). Lignin variability in plant cell walls: Contribution of new models. Plant Sci..

[4] Ragauskas AJ (2014). Lignin valorization: Improving lignin processing in the biorefinery. Science.

[5] Balakshin MY (2021). New opportunities in the valorization of technical lignins. ChemSusChem.

[6] Schutyser W (2018). Chemicals from lignin: an interplay of lignocellulose fractionation, depolymerisation, and upgrading. Chem. Soc. Rev..

[7] Sun R-C, Samec JSM, Ragauskas AJ (2020). Preface to special issue of ChemSusChem on lignin valorization: From theory to practice. ChemSusChem.

[8] Subbotina E (2021). Oxidative cleavage of C–C bonds in lignin. Nat. Chem..

[9] Sun R-C (2020). Lignin source and structural characterization. ChemSusChem.

[10] Bertella S, Luterbacher JS (2020). Lignin functionalization for the production of novel materials. Trends Chem..

[11] Figueiredo P, Lintinen K, Hirvonen JT, Kostiainen MA, Santos HA (2018). Properties and chemical modifications of lignin: Towards lignin-based nanomaterials for biomedical applications. Prog. Mater. Sci..

[12] Wu X (2018). Solar energy-driven lignin-first approach to full utilization of lignocellulosic biomass under mild conditions. Nat. Catal..

[13] Shu F (2021). Biological activities and emerging roles of lignin and lignin-based products─A review. Biomacromolecules.

[14] Zhang B (2022). Transition-metal-free synthesis of pyrimidines from lignin β-O-4 segments via a one-pot multi-component reaction. Nat. Commun..

[15] Li W, Chen Z, Yu H, Li J, Liu S (2021). Wood-derived carbon materials and light-emitting materials. Adv. Mater..

[16] Li J (2022). Lignin: a sustainable photothermal block for smart elastomers. Green. Chem..

[17] Yuan J (2021). Sustainable afterglow materials from lignin inspired by wood phosphorescence. Cell Rep. Phys. Sci..

[18] Miao Q (2017). Molecular afterglow imaging with bright, biodegradable polymer nanoparticles. Nat. Biotechnol..

[19] Li Y, Gecevicius M, Qiu J (2016). Long persistent phosphors—from fundamentals to applications. Chem. Soc. Rev..

[20] Baryshnikov G, Minaev B, Ågren H (2017). Theory and calculation of the phosphorescence phenomenon. Chem. Rev..

[21] Xu S, Chen R, Zheng C, Huang W (2016). Excited state modulation for organic afterglow: materials and applications. Adv. Mater..

[22] Zhao W, He Z, Tang BZ (2020). Room-temperature phosphorescence from organic aggregates. Nat. Rev. Mater..

[23] Wang H, Shi H (2021). Lignin rebirth enables sustainable afterglow emission. Matter.

[24] Sun Y (2020). Ultralong lifetime and efficient room temperature phosphorescent carbon dots through multi-confinement structure design. Nat. Commun..

[25] Wu Z, Nitsch J, Marder TB (2021). Persistent room-temperature phosphorescence from purely organic molecules and multi-component systems. Adv. Opt. Mater..

[26] Sun S, Wang J, Ma L, Ma X, Tian H (2021). A universal strategy for organic fluid phosphorescence materials**.. Angew. Chem. Int.

[27] Gao R, Yan D (2017). Layered host–guest long-afterglow ultrathin nanosheets: high-efficiency phosphorescence energy transfer at 2D confined interface. Chem. Sci..

[28] Hamzehpoor E, Perepichka DF (2020). Crystal engineering of room temperature phosphorescence in organic solids. Angew. Chem. Int. Ed..

[29] Zhang G, Palmer GM, Dewhirst MW, Fraser CL (2009). A dual-emissive-materials design concept enables tumour hypoxia imaging. Nat. Mater..

[30] Su Y (2018). Ultralong room temperature phosphorescence from amorphous organic materials toward confidential information encryption and decryption. Sci. Adv..

[31] Cai S (2021). Ultralong organic phosphorescent foams with high mechanical strength. J. Am. Chem. Soc..

[32] Jinnai K, Kabe R, Lin Z, Adachi C (2022). Organic long-persistent luminescence stimulated by visible light in p-type systems based on organic photoredox catalyst dopants. Nat. Mater..

[33] Chen C (2020). Structure–property–function relationships of natural and engineered wood. Nat. Rev. Mater..

[34] Ma R, Guo M, Zhang X (2014). Selective conversion of biorefinery lignin into dicarboxylic acids. ChemSusChem.

[35] Cronin DJ, Zhang X, Bartley J, Doherty WOS (2017). Lignin depolymerization to dicarboxylic acids with sodium percarbonate. ACS Sustain. Chem. Eng..

[36] Cai Z (2019). Selective Production of diethyl maleate via oxidative cleavage of lignin aromatic unit. Chem.

[37] An Z (2015). Stabilizing triplet excited states for ultralong organic phosphorescence. Nat. Mater..

[38] Usha R, Ramasami T (2002). Effect of hydrogen-bond-breaking reagent (urea) on the dimensional stability of rat tail tendon (RTT) collagen fiber. J. Appl. Polym. Sci..

[39] Liu X (2021). Hydrogen-binding-initiated activation of O-H bonds on a nitrogen-doped surface for the catalytic oxidation of biomass hydroxyl compounds. Angew. Chem. Int. Ed..

[40] Xia Q (2021). In situ lignin modification toward photonic wood. Adv. Mater..

[41] Zhang Y, Naebe M (2021). Lignin: A review on structure, properties, and applications as a light-colored UV absorber. ACS Sustain. Chem. Eng..

