# Supplementary material for: Structural materials with afterglow room temperature phosphorescence activated by lignin oxidation
Source: Nat Commun. 2022 Sep 20;13:5508. doi: 10.1038/s41467-022-33273-1 (PMC9489714; doi:10.1038/s41467-022-33273-1)
Supplement: Supplementary file 1 — Supplementary Information [file 41467_2022_33273_MOESM1_ESM.pdf]

Supplementary Information

Structural materials with afterglow room temperature phosphorescence activated by lignin oxidization

Keliang Wan[1,5], Bing Tian[1,5], Yingxiang Zhai[1,5], Yuxuan Liu[2,5], He Wang[3], Shouxin Liu[1], Shujun Li[1], Wenpeng Ye[3], Zhongfu An[3,*], Changzhi Li[2,*], Jian Li[1], Tony D. James[4], and Zhijun Chen[1,*]

[1]Engineering Research Center of Advanced Wooden Materials and Key Laboratory of Bio-based Material Science & Technology, Northeast Forestry University, Ministry of Education, Harbin, China. [2]CAS Key Laboratory of Science and Technology on Applied Catalysis, Dalian Institute of Chemical Physics, Chinese Academy of Sciences, Dalian, China. [3]Key Laboratory of Flexible Electronics & Institute of Advanced Materials, Nanjing Tech University, Nanjing, China. [4]Department of Chemistry, University of Bath, Bath BA2 7AY, United Kingdom.
[5]These authors contributed equally to this work.
*e-mail: chenzhijun@nefu.edu.cn; licz@dicp.ac.cn; iamzfan@njtech.edu.cn

Materials

Lignin used here were the lignosulfonate (containing 8 wt. % Na and 4 wt. % reducing sugars), purchased from Sigma-Aldrich (Shanghai, China). All the wood samples were purchased from Taobao (Alibaba, Hangzhou, China). Alkali lignin were prepared as follows: Poplar wood powders (100 g) was mixed with NaOH aqueous solution (10% w/w, 600 mL). The mixture was maintained at 120 ^{o}C for 1.5 h. After that, the solid was removed via filtration. As-obtained black liquor was acidified (HCl solution, 1 M) until the pH was 2. The solid precipitation was dried to give alkali lignin[1]. NaOH (>96%), H_2O_2 solution (30% w/w), urea (>99%), HCl solution (36-38% w/w) and dichloromethane (>99.5%) were purchased from Kermel Chemical Industry (Tianjin, China). Ethyl alcohol (>99.7%) and acetonitrile (>99%) were purchased from Aladdin (Shanghai, China). Deionized water was produced using a Smart-RO ultrapure water system (Hitech Instruments Co., Ltd., Shanghai, China). Components for the custom-built apparatus were purchased from Taobao (Alibaba, Hangzhou, China).

Characterization

Fluorescence spectra were recorded using an LS-55 fluorescence spectrophotometer (PerkinElmer, Inc., Waltham, MA, USA), equipped with a 120 W xenon lamp as the excitation source. UV-Vis absorption spectra were recorded using a TU-1901 UV-Vis double-beam spectrophotometer (Persee General Instrument Co., Ltd., Beijing, China). Phosphorescence spectra and lifetime decay curves were recorded using an FLS1000 photoluminescence spectrometer (Edinburgh Instruments, Livingston, UK), equipped with a xenon lamp and a microsecond flashlamp (detector: photomultiplier tube, 200 nm $< \lambda <$ 1700 nm). Temperature was controlled using an OX135QX cryostat (Oxford Instruments plc, Abingdon, UK). HRMS spectra was recorded on a Agilent 6540 Q-TOF mass

spectrometer; 2D HSQC NMR spectra was recorded on a Bruker 700 MHz NMR spectrometer with TMS as the internal standard.

Theoretical Simulation

A three-dimensional periodic boundary condition[2] was applied to the whole system. 64 A molecules and 160 B molecules were introduced into the supercell with lattice parameters **a** = **b** = **c** = 45 Å, $\alpha = \beta = \gamma = 90°$. Then, MD calculations were performed by a classical molecular dynamic simulation method employing a modified cff91 force field[3]. A charge equilibration (QEq) method[4] was used to calculate the atomic charges of the layer. Other forcefield parameters for the anions were referred to the cff91 forcefield[5]. After geometry optimization was applied to the initial model and the optimized lattice parameters **a** = 25.2 Å, **b** = 44.7 Å, **c** = 30.1 Å, MD simulations were performed in an isothermal-isobaric (NPT) ensemble with a typical thermodynamic temperature of 273 K and typical pressures of 0.1 MPa. The Andersen method[6] and Berendsen method[7] were used to control temperature and pressure, respectively. The total simulation time was 500 ps with a simulation time step of 0.1 fs. The simulations were performed using the Forcite module in the Material Studio software package[8].

The spin-orbit couplings between singlet ant triplet states were calculated at TD-DFT / PBE0 / ma-def2-TZVP level. The calculations were performed using the ORCA package[9].

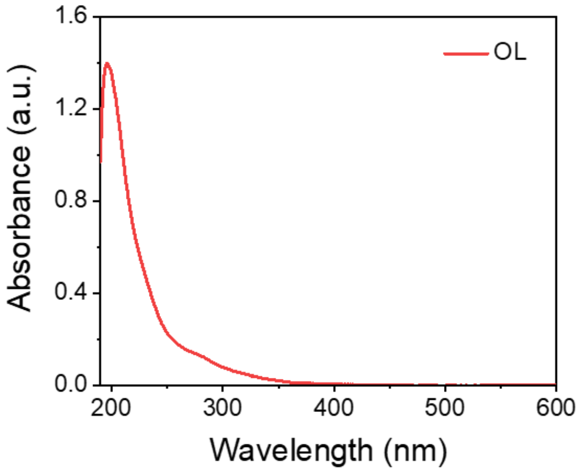

Supplementary Fig. 1. UV-Vis absorbance of OL.

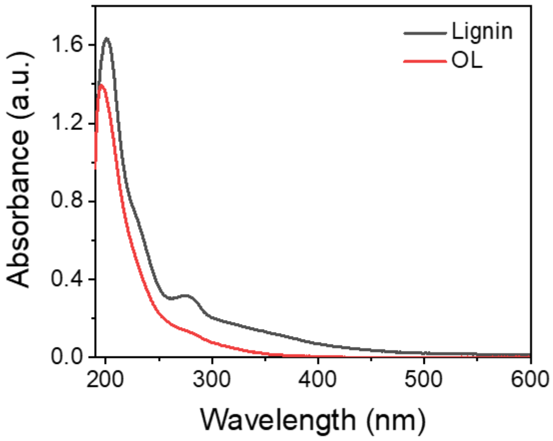

Supplementary Fig. 2. UV-Vis absorbance of Lignin and OL.

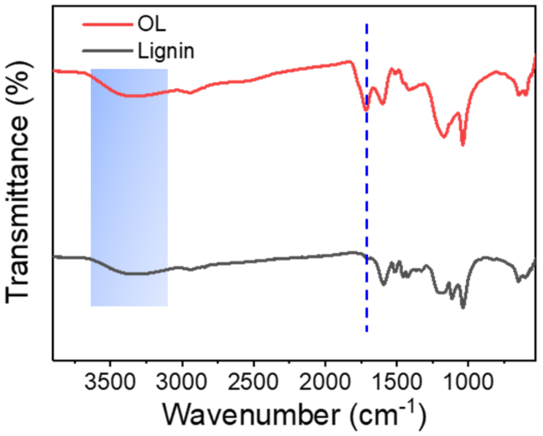

Supplementary Fig. 3. FT-IR spectra of lignin and OL.

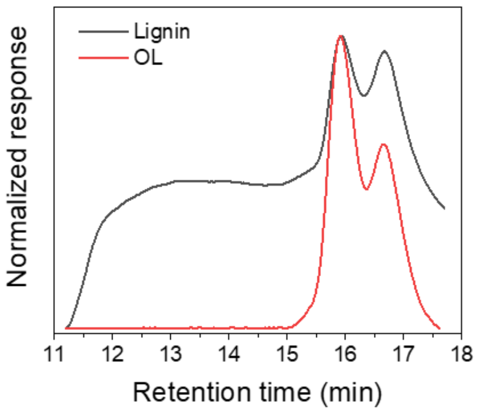

Supplementary Fig. 4. GPC traces of lignin and OL. (Lignin: Mn = 2102, Mw = 11541, PD = 5.49; OL: Mn = 1382, Mw = 1588, PD=1.14). Gel permeation chromatography (GPC) was carried out with Agilent PL-GPC50 equipped with refractive index detector. The GPC column was double PL gel aquagel-OH Mixed-M with flow rate 1.0 mL min^{-1}, using aqueous solution (0.2 M sodium nitrate ($NaNO_3$), 0.1 M monosodium phosphate NaH_2PO_4, buffered to pH 7.0) as the mobile phase. A calibration curve was obtained using Polyethylene glycol (PEG) (Agilent Technologies Inc., Palo Alto, CA, USA) as a standard.

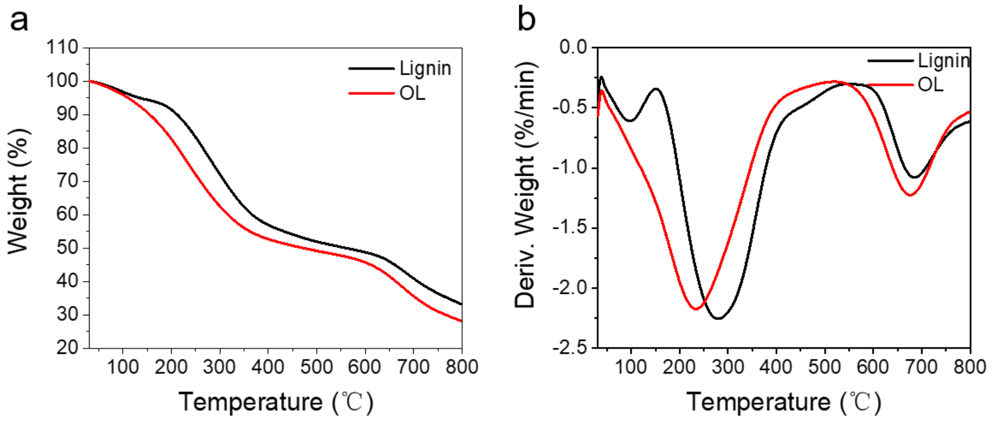

Supplementary Fig. 5. (a) TGA of Lignin and OL. (b) DTG of Lignin and OL. The measurements were conducted in N_2 atmosphere.

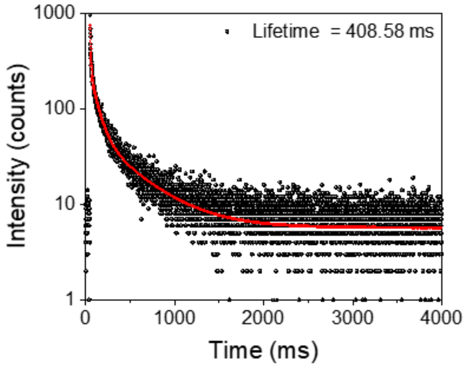

Supplementary Fig. 6. Lifetime of OL made from lignin, excitation wavelength = 365 nm.

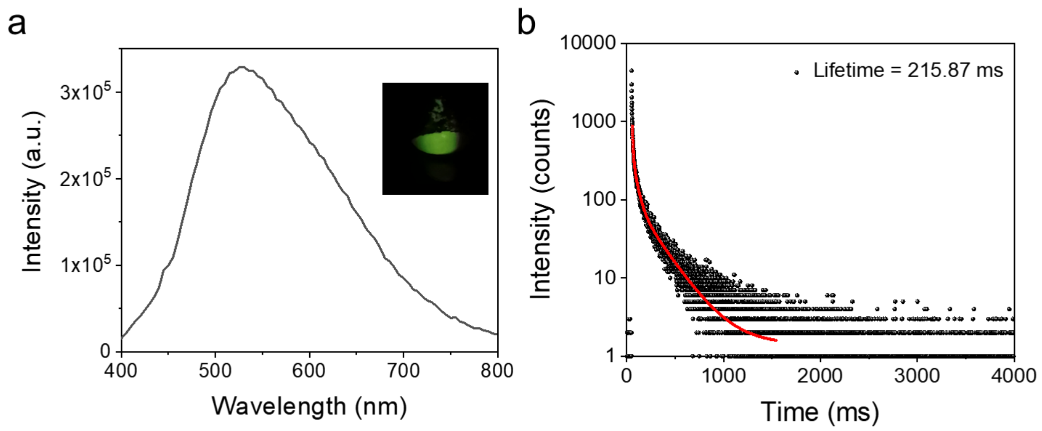

Supplementary Fig. 7. RTP intensity (**a**) and lifetime (**b**) of OL made from alkali lignin. Inset: afterglow emission of OL after switching the UV sources off, excitation wavelength = 365 nm.

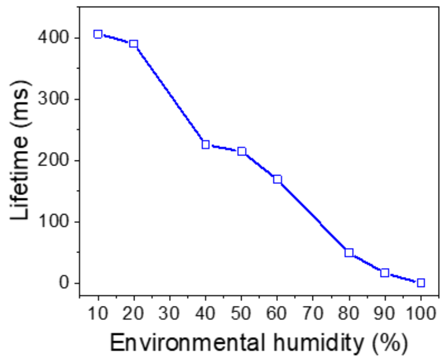

Supplementary Fig. 8. RTP lifetime of OL upon exposure to different humidity.

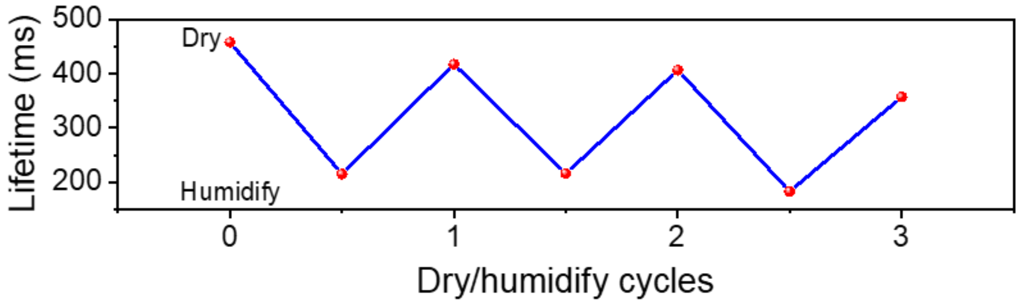

Supplementary Fig. 9. RTP lifetime of OL upon recyclable treatment of humidity (30%) and drying (120 °C).

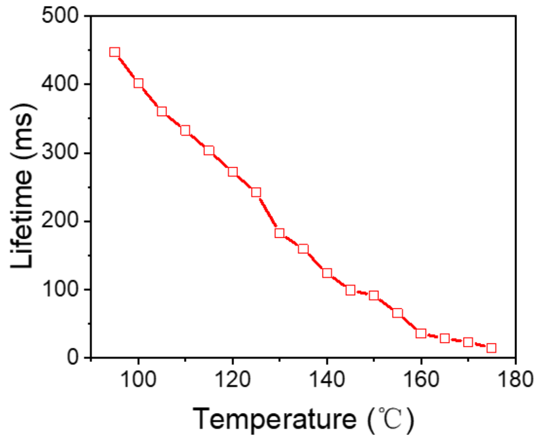

Supplementary Fig. 10. RTP lifetime of OL upon exposure to different temperature for 90 min.

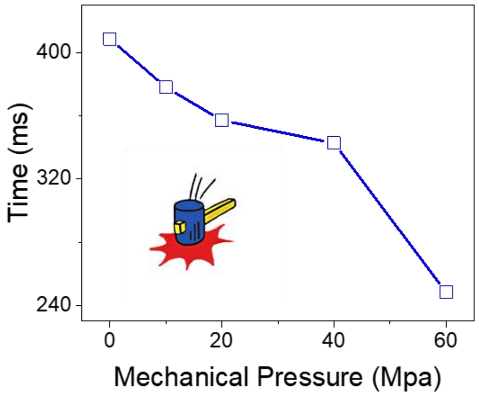

Supplementary Fig. 11. RTP lifetime of OL upon mechanical treatment.

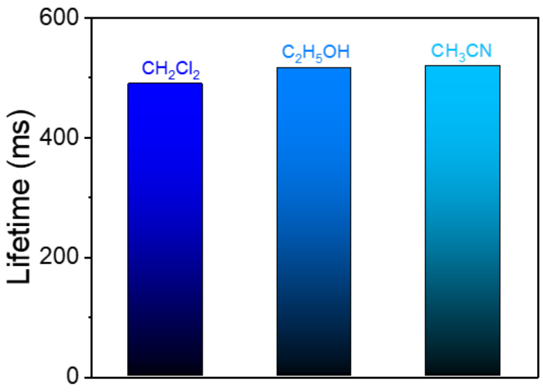

Supplementary Fig. 12. RTP lifetime of OL in different organic solvent.

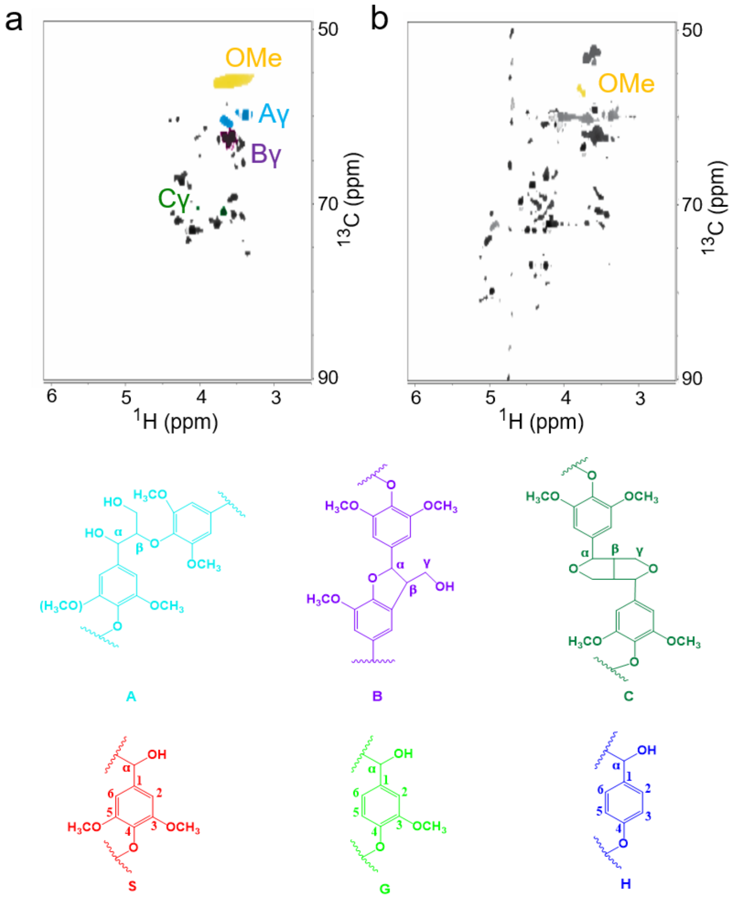

Supplementary Fig. 13. 2D HSQC NMR of (**a**) lignin and (**b**) OL in the fatty region.

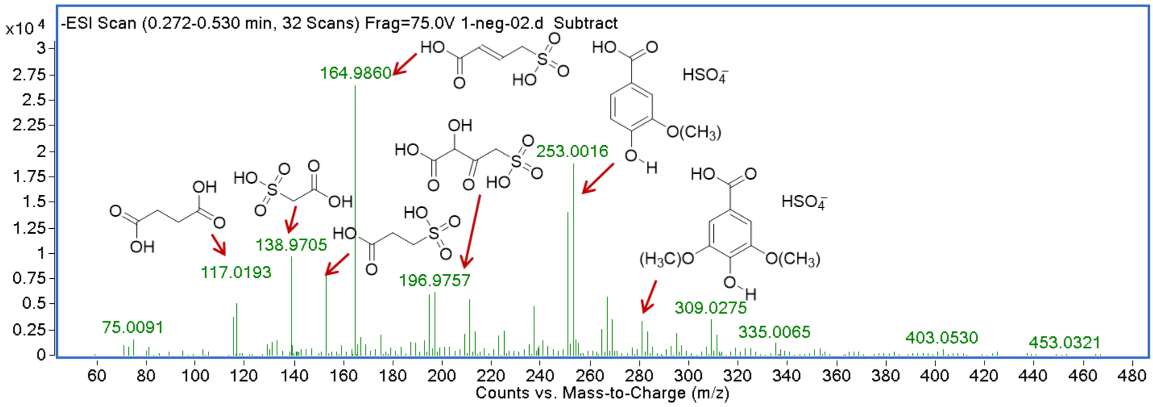

Supplementary Fig. 14. HRMS spectra of OL.

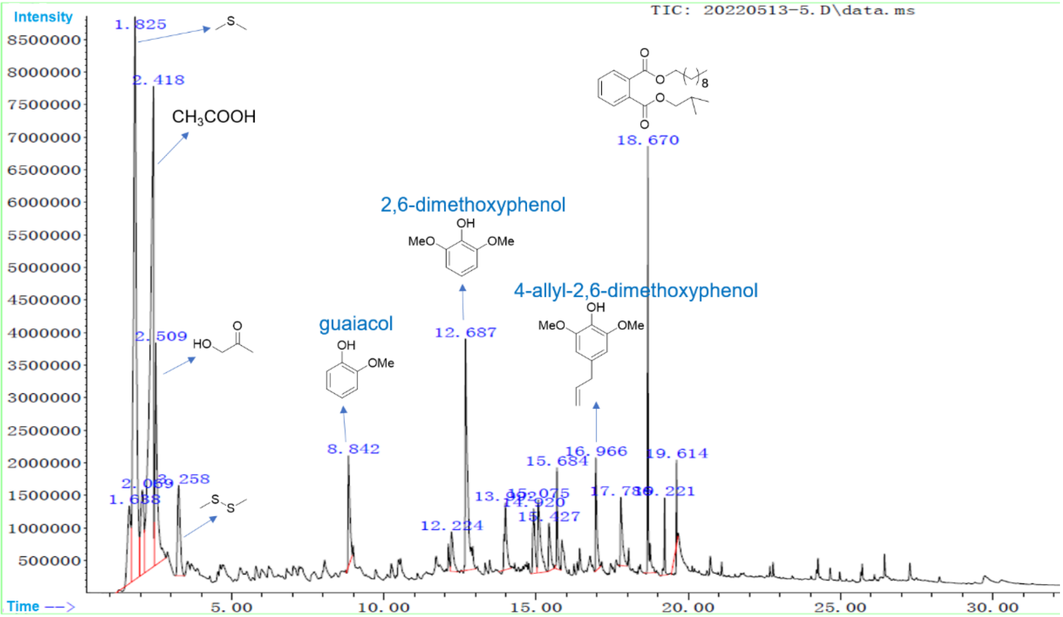

Supplementary Fig. 15. Py-GC-MS analysis of lignin.

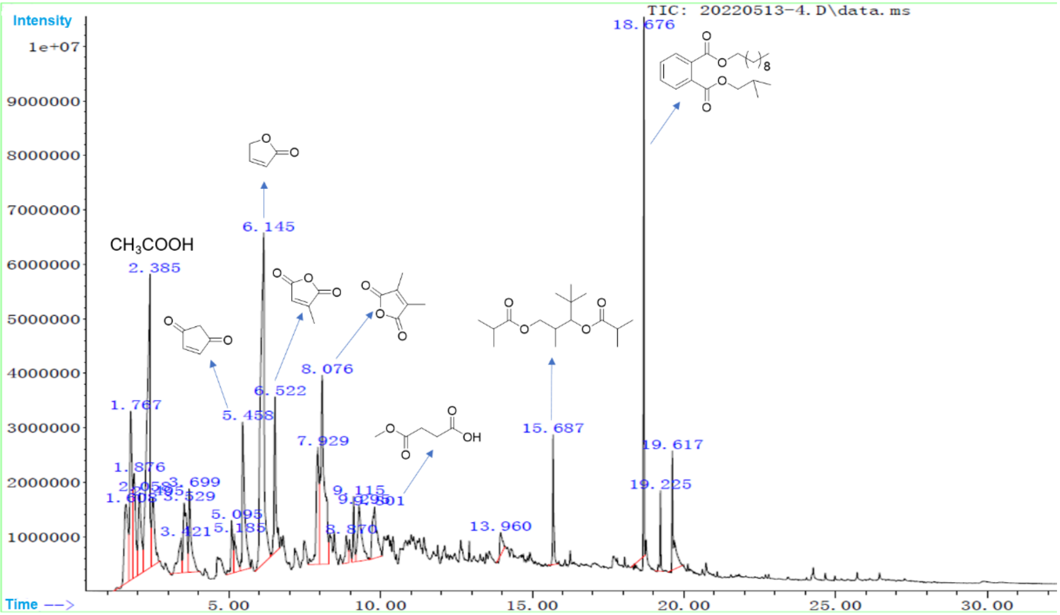

Supplementary Fig. 16. Py-GC-MS analysis of OL.

Fast pyrolysis was performed by Py-GC-MS. Experiments were carried out with a Pyroprobe 5000 Series (CDS Analytical Inc.) with direct connection to a Agilent 7890A gas chromatograph (GC) equipped with a Agilent 5975C mass spectrometer (MS). About 0.5 mg samples were prepared and introduced into the Pyroprobe in quartz tubes. The pyroprobe was initially set and held for 5 s at 50 ^{o}C, and then ramped at 1 ^{o}C/20 ms to a final temperature of 550 ^{o}C and held for 20 s.

The GC separation of pyrolysis vapors was performed with a 30 m × 0.25 mm × 1.0 μm VF1701ms column from Agilent, with helium carrier gas flow of 1 mL/min. The GC inlet was 280 ^{o}C and a split ratio of 25:1 was used. The oven was programmed to start at 40 ^{o}C. It was held at 40 ^{o}C for 5 min and then heated at 10 ^{o}C/min to a final temperature of 280 ^{o}C and kept for 20 min. Mass spectra were recorded under electron ionization (70 eV). Peak identification was carried out with the NIST mass spectral library and literature.

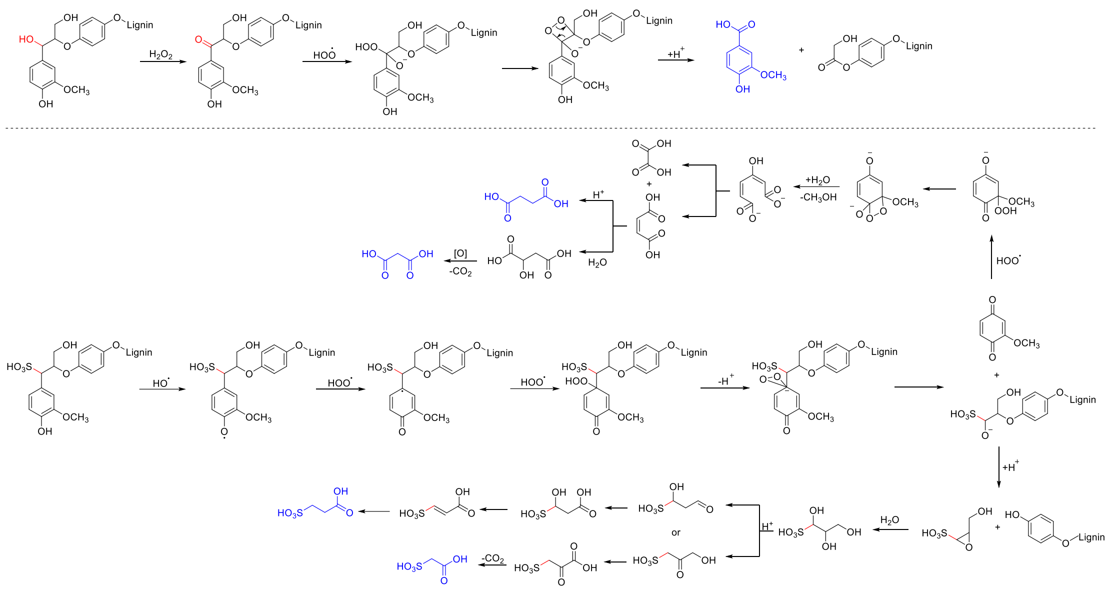

Supplementary Fig. 17. Proposed reaction route from lignin to OL via oxidation.

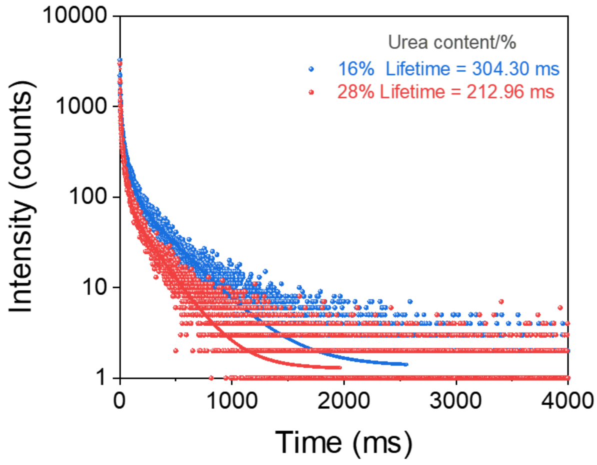

Supplementary Fig. 18. RTP lifetime of OL upon addition of urea, excitation wavelength = 365 nm.

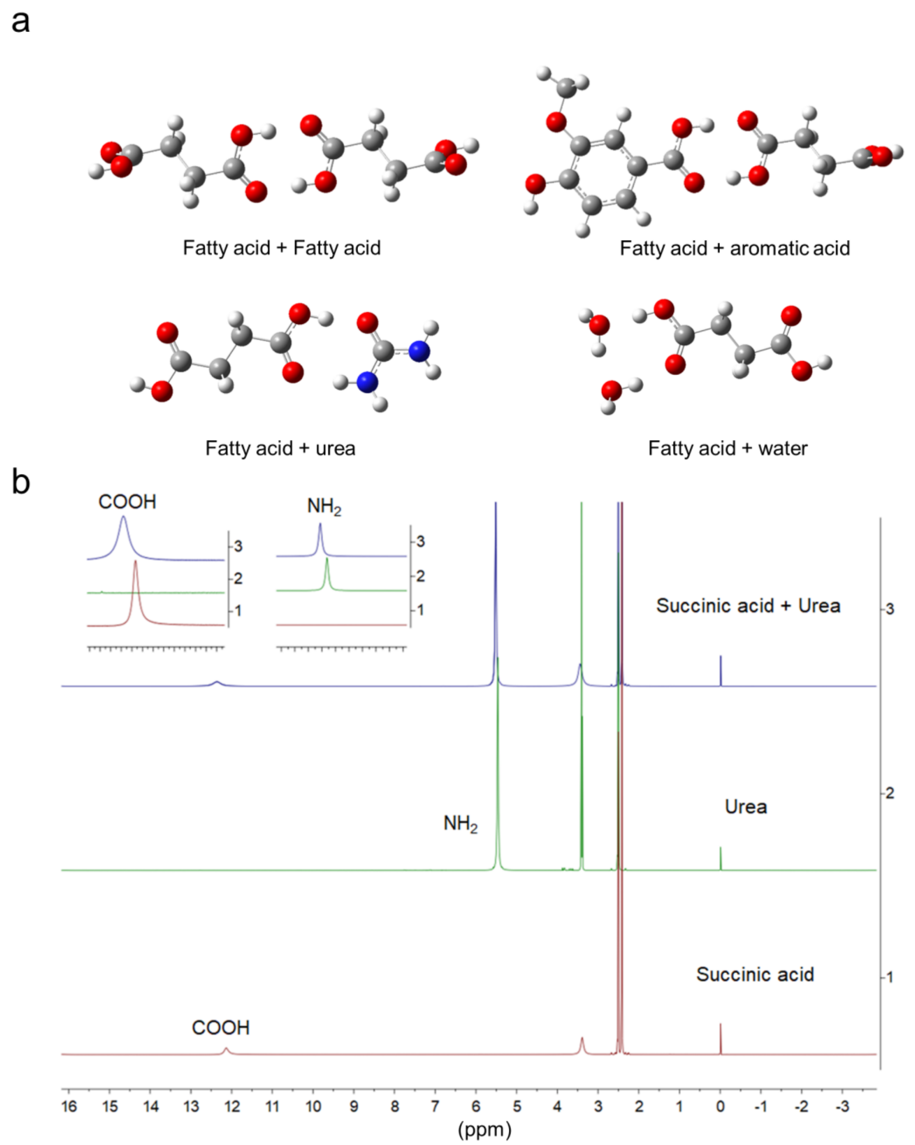

Supplementary Fig. 19. a) Simulation of interaction between fatty acid/fatty acid, fatty acid/aromatic acid, fatty acid/urea and fatty acid/water. b) ^{1}H NMR of succinic acid, urea and succinic acid/urea in DMSO-d_6.

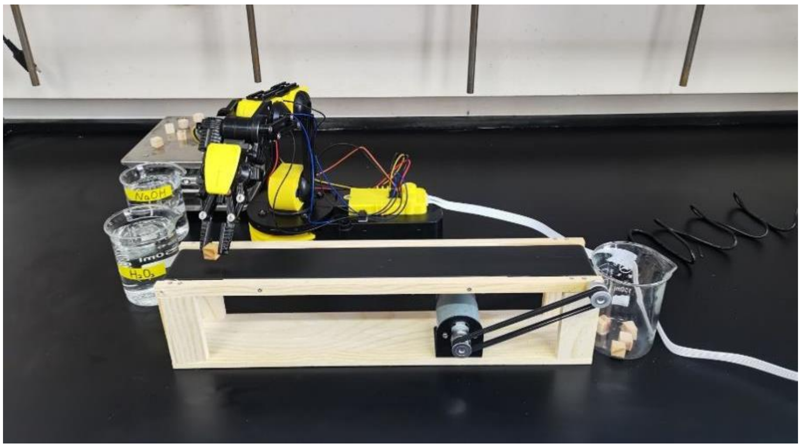

Supplementary Fig. 20. Digital images for the automatic manufacturing of RTP wood.

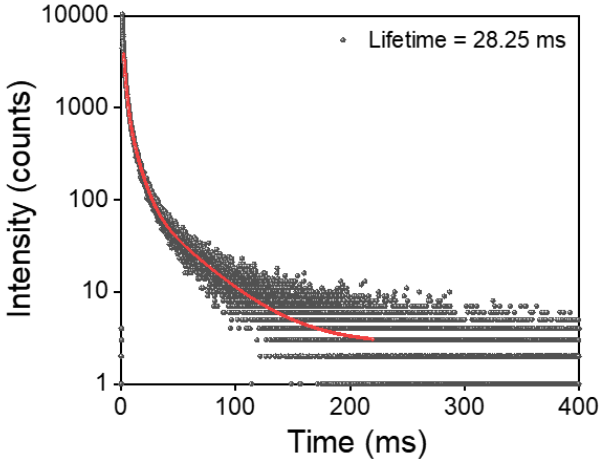

Supplementary Fig. 21. Phosphorescence lifetime of natural Basswood.

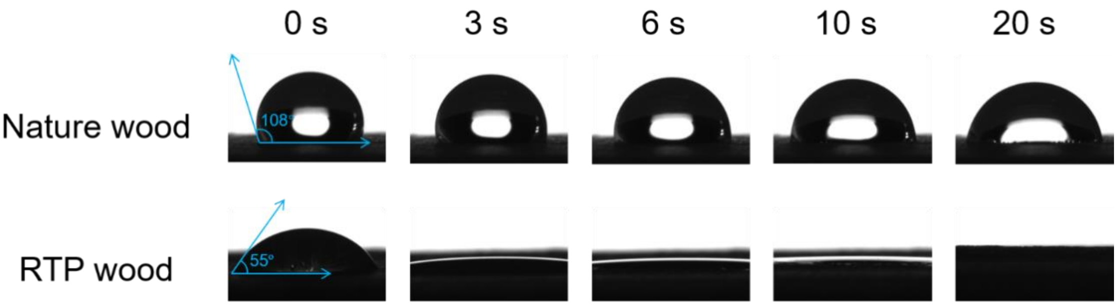

Supplementary Fig. 22. Contact angle of nature wood and RTP wood.

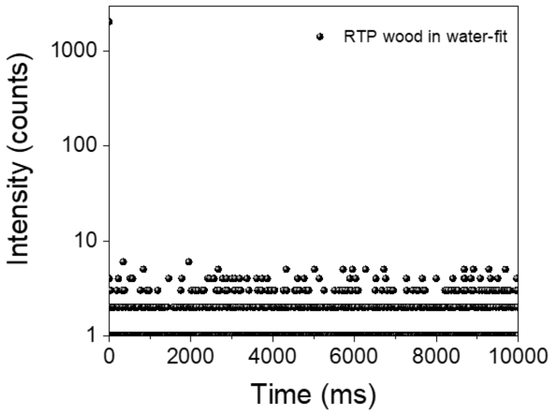

Supplementary Fig. 23. RTP lifetime of RTP wood in water.

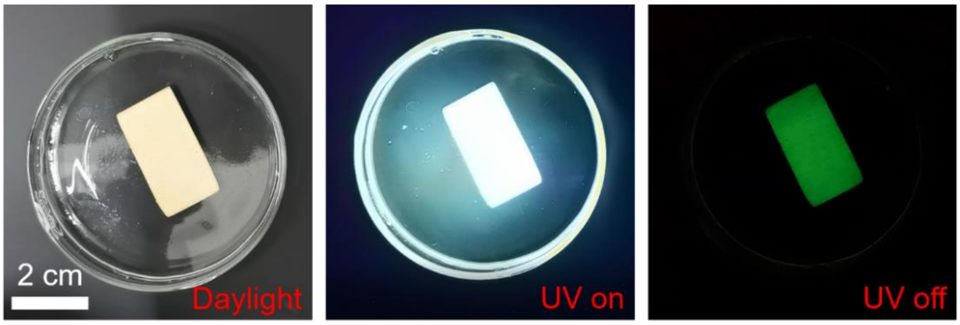

Supplementary Fig. 24. Digital images of RTP wood decorated with wood wax upon UV irradiation immersed in water.

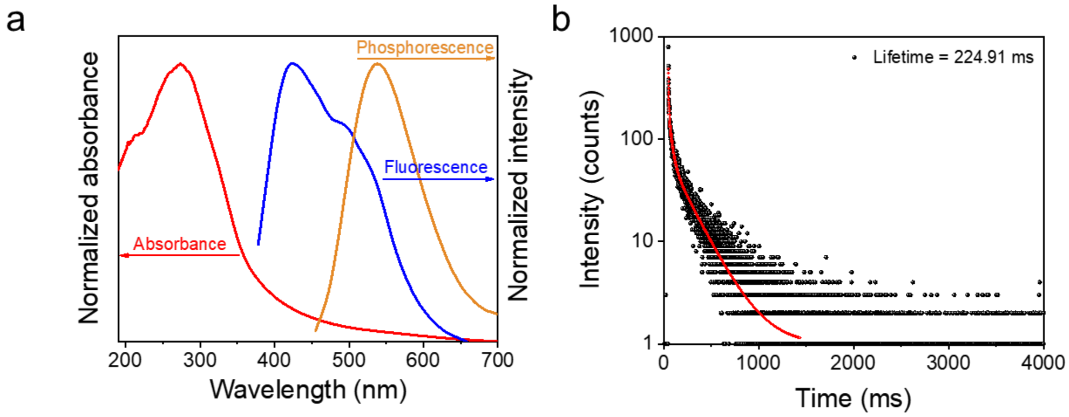

Supplementary Fig. 25. (**a**) Absorbance, fluorescence and phosphorescence of RTP wood made from Schima Superb. (**b**) Phosphorescence lifetime of RTP wood made from Schima Superb.

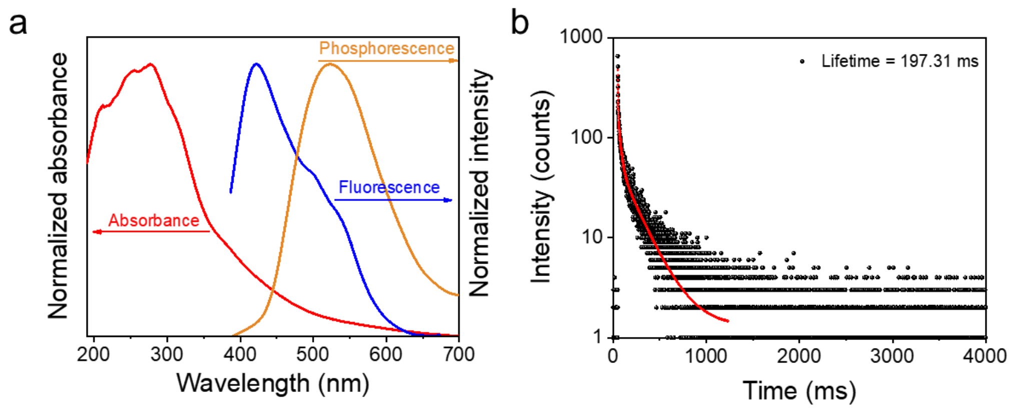

Supplementary Fig. 26. (**a**) Absorbance, fluorescence and phosphorescence of RTP wood made from Rubber wood. (**b**) Phosphorescence lifetime of RTP wood made from Rubber wood.

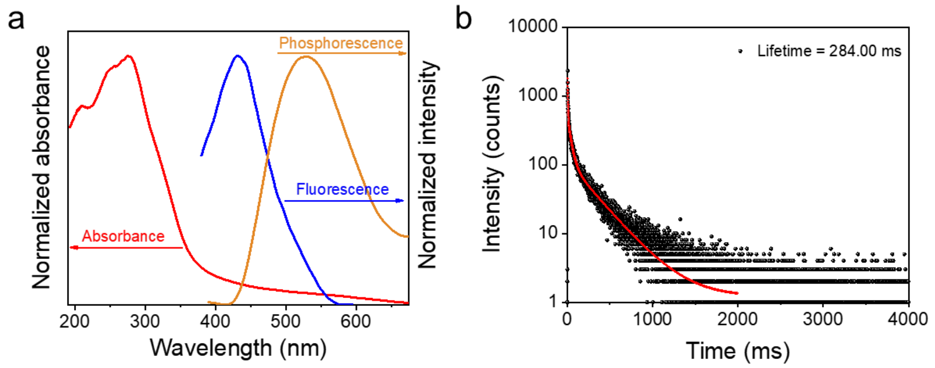

Supplementary Fig. 27. (**a**) Absorbance, fluorescence and phosphorescence of RTP wood made from Peach wood. (**b**) Phosphorescence lifetime of RTP wood made from Peach wood.

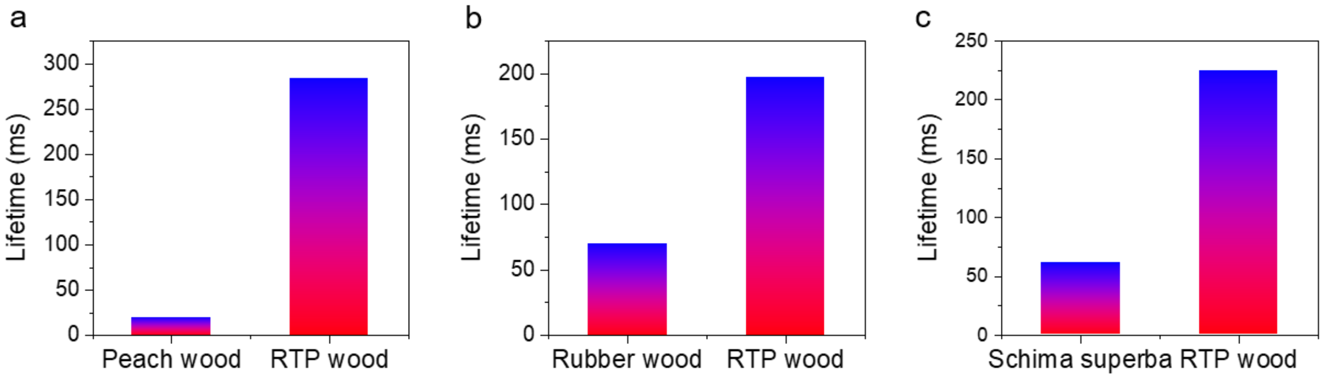

Supplementary Fig. 28. (a) Comparison between lifetime of natural Peach wood and RTP wood made from Peach wood. (b) Comparison between lifetime of natural Rubber wood and RTP wood made from Rubber wood. (c) Comparison between lifetime of natural Schima superba and RTP wood made from Schima superba.

References

1. Zhao X, et al. Melanin-inspired design: preparing sustainable photothermal materials from lignin for energy generation. *ACS Appl. Mater. Interfaces* **13**, 7600-7607 (2021).
2. Leach, A. Molecular modelling: principles and applications. *Pearson education* (2001).
3. Yan, D. *et al.* In situ polymerization of the 4-vinylbenzenesulfonic anion in Ni−Al−layered double hydroxide and its molecular dynamic simulation. *J. Phys. Chem. A* **112**, 7671-7681 (2008).
4. Rappe, A. & Goddard, W. Charge equilibration for molecular dynamics simulations. *J. Phys. Chem.* **95**, 3358-3363 (1991).
5. Maple, J. *et al.* Derivation of class II force fields. I. methodology and quantum force field for the alkyl functional group and alkane molecules. *J. Comput. Chem.* **15**, 162-182 (1994).
6. Andersen, H. Molecular dynamics simulations at constant pressure and/or temperature. *J. Chem. Phys.* **72**, 2384-2393 (1980).
7. Berendsen, H., Postma, J., van Gunsteren, W. & Hermans, J. Interaction models for water in relation to protein hydration. in: pullman B (ed). *Intermolecular Forces: Proceedings of the Fourteenth Jerusalem Symposium on Quantum Chemistry and Biochemistry Held in Jerusalem, Israel, April 13–16, 1981*. Springer Netherlands: Dordrecht, 1981, pp 331-342.
8. Module D. MS Modeling, Version 2.2. *Accelrys Inc: San, Diego, CA* 2003.
9. Neese F. Software update: the ORCA program system, version 4.0. *WIREs Computational Molecular Science* 2018, **8**(1)**:** e1327.
