# Supplementary material for: Structural materials with afterglow room temperature phosphorescence activated by lignin oxidation
Source: Nat Commun. 2022 Sep 20;13:5508. doi: 10.1038/s41467-022-33273-1 (PMC9489714; doi:10.1038/s41467-022-33273-1)
Supplement: Supplementary file 2 — Description of Additional Supplementary Files [file 41467_2022_33273_MOESM2_ESM.pdf]

Description of Additional Supplementary Files

File Name: Supplementary Movie 1
Description: The automatic manufacturing line for RTP wood.

File Name: Supplementary Movie 2
Description: Recognizing the 2-D code using a smartphone.
